# Sustainable Economic Development, Digital Payment, and Consumer Demand: Evidence from China

**DOI:** 10.3390/ijerph19148819

**Published:** 2022-07-20

**Authors:** Rui Zhou

**Affiliations:** School of Economics, Fudan University, Shanghai 200433, China; zhourui@sina.cn

**Keywords:** sustainable economic development, digital payment, electronic payment, consumer demand, COVID-19 pandemic

## Abstract

In this era, the global COVID-19 pandemic has hit the economy hard. In the context of great challenges to sustainable economic development, it is of great practical significance to study how digital payment can promote consumer demand and sustainable economic development. From the perspective of sustainable economic development, this paper selects panel data of various provinces in China from 2011 to 2020 to test the correlation between digital payment and consumer demand by constructing econometric models and selecting relevant indicators, so as to reveal the impact of digital payment on consumer demand and sustainable economic development. Research shows that: (1) in the context of the COVID-19 pandemic, digital payments play a special and very important role in promoting household consumption and sustainable economic development; (2) the empirical results show that digital payment has a significant positive impact on consumer demand, which indicates that digital payment has an obvious promotion effect on consumer demand; (3) further research shows that the impact of digital payment on consumer demand has obvious heterogeneity. From the perspective of regional differences, digital payment has a significant positive impact on consumer demand in the eastern and western regions, while the impact is not obvious in the northeast and central regions, even though it also has a positive impact. From the perspective of urban-rural differences, digital payment has a significant impact on consumer demand in both urban and rural areas, and this impact is greater in rural areas than in urban areas. However, from the perspective of development stage, the stage characteristics of digital payment’s impact on consumer demand in each region are not obvious, which may be caused by the short sample range. In addition, this paper also puts forward relevant suggestions for other countries to learn from.

## 1. Introduction

In the context of the huge impact of the COVID-19 pandemic on the economy, how to deal with the negative impact of COVID-19 and achieve sustainable economic development has become an important issue of concern to all countries in the world. In June 2022, the World Bank published an article titled “COVID-19 Pandemic drives A Surge in Global Digital Payment Use”, which noted that financial inclusion was expanding globally during the pandemic, driving a significant growth in digital payments. Digital payment refers to the digital payment method achieved by means of communication technology, artificial intelligence, and information technology with the help of computer, intelligent equipment, and other hardware equipment. These digital means mainly include electronic payment, electronic money, and digital money. In this respect, the meaning of electronic payment is relatively broad. Electronic money and digital money are the premise of electronic payment, and their payment function also needs electronic payment to realize, so electronic payment represents digital payment to a large extent.

With the rapid development of payment technology in recent years, many scholars begin to pay attention to digital payment, and a large number of studies focus on the willingness and behavior of digital payment, and the impact of digital payment on the major sectors of industry [1,2,3,4,5,6,7,8,9,10]. There are also studies that show that digital payment changes the public’s consumption behavior and affects consumer demand [11,12,13,14,15,16,17]. However, there is little research on the relationship between digital payment and consumer demand. In China, data from China’s National Bureau of Statistics show that since 2015, consumption has contributed more than 58% to China’s economic growth. There is no doubt that consumption has become the number one driver of China’s three economic engines. In the meantime, it can be said that payment, as an important part of the consumption process, has a direct or indirect impact on consumer demand. However, does digital payment development have an impact on consumer demand? How does it impact it? So far, there is no consensus and more controversy in the theoretical circle, and it is still an outstanding issue. More importantly, in-depth exploration of this issue is not only conducive to a more objective understanding of the impact of the evolution of payment methods on consumption and the real economy, but also conducive to the development of the payment system in the future. 

The main purpose of this study is to study how digital payment can promote consumer demand and sustainable economic development in the context of the COVID-19 pandemic, the severe impact on global economy, and the great challenges to sustainable economic development. Therefore, from the perspective of sustainable economic development, based on consumer demand theory, inclusive finance theory, and technology acceptance theory, this paper tries to construct an econometric model. In this paper, panel data of various provinces in China from 2011 to 2020 are selected, indicators related to digital payment and consumer demand are selected to test the correlation between digital payment and consumer demand, so as to reveal the impact of digital payment on consumer demand and sustainable economic development, and put forward relevant policy suggestions.

The contribution of this paper is mainly reflected in the following aspects: (1) the research perspective is novel, and the impact of digital payment on consumer demand is studied from the perspective of sustainable economic development; (2) in the context of the impact of COVID-19, it proposes how to drive consumer demand through digital payment and promote sustainable economic development; (3) different from previous theoretical studies focusing on factors influencing consumer demand, this paper lays more emphasis on empirical analysis and reveals the main factors influencing consumer demand through empirical tests; and (4) this paper also tests the influence of digital payment on urban residents’ consumption, rural residents’ consumption and the per capita consumption of all residents, and conducts a heterogeneity test and comparative analysis of residents’ consumption of different types, different regions and different stages.

Next, this paper mainly includes the following parts. The second part is a literature review, the third part is the study design, the fourth part is the results and discussion, and the fifth part is the conclusion and implications.

## 2. Literature Review 

As the traditional consumption theory is the theoretical basis for constructing the consumption behavior model including digital payment method, this part will sort out and evaluate the relevant literature from four aspects: COVID-19 pandemic and sustainable economic development, intention and behavior of using digital payment, consumption behavior and consumer demand, and influence of digital payment on consumer demand.

### 2.1. COVID-19 Pandemic and Sustainable Economic Development

Deliu [18] argues that the world is currently in the midst of a pandemic crisis that will affect sustainable economic development and that multilateral organizations and citizens of countries urgently need to develop strategies to combat, respond to, mitigate, and protect their societies and economies during and after the socio-economic crisis. Chen’s [19] research results show that given the enormous impact that the COVID-19 pandemic had on China’s economy, helping companies to revitalize post-pandemic economic activities promptly is a priority for the whole society. This necessitates the smooth circulation of production-factors among different economic entities, departments, and regions. The pandemic’s huge impact on the economy is evident in the severely hampered flow of these factors, including labor, materials, and capital. Therefore, using data and digital technology, combined with a contact-free allocation of labor, capital, and materials, to accelerate the flow of production factors is critical to the post-pandemic economy’s restoration. Such a policy can not only provide a short-term stimulus but also momentum for China’s mid- and long-term sustainable economic development. Li et al. [20] discuss the impact of different economic indicators on economic stability, including honest leadership, improved infrastructure, revenue generation, and CPEC taking into account the double mediating role of environmental sustainability and sustainable development, while considering the latest COVID-19 situation. The results reveal that honest leadership, improved infrastructure, revenue generation, and CPEC have a positive nexus with economic stability. Despite the severe impact of COVID-19 on the country’s economy, the economic corridor plays a vital role in stabilizing the state’s economy and supports all those related to this phenomenal project either directly or indirectly. Nundy [21] believes that a global pandemic occurred in 2020 because of COVID-19. Compared to the pre-pandemic period, the outbreak has adversely affected human life, the economy, environment, energy, and transportation sectors. As of now, the COVID-19 epidemic continues to grow in some regions, causing anxiety and disruption. Despite these causes, impacts, and recovery plans, the sustainable development goals will still be heavily affected if they are to be achieved by 2030, and cooperative support from all countries can only help in the meantime. Ahmed and Sarkodie’s [22] research results show that despite the unprecedented macroeconomic policy supporting the decline in global FDI, GDP, and trade amidst COVID-19 pandemic, economic policies will not be sustainable in the long term if restrictions and containment measures are not eased. It is observed that many large economies are at risk of gross government debts, which may hamper sustainable economic development. Yin et al. [23] provide insights into the importance of ESs to the SDGs and the ways to integrate ESs into socio-economic development to promote the SDG achievement after the pandemic. Ecosystem services (ESs), defined as the contributions of ecosystems to human well-being, underpin the achievement of SDGs. Study results found that ESs benefited all SDGs, yet man-made pressures led to the degradation of ecosystems and their services. Zhou et al.’s [24] research shows that, in the context of the COVID-19 pandemic, the improvement of ESG performance of listed companies can enhance the market value of the company, thus promoting the sustainable development of the company. Vavoura and Vavouras [25] show that, taking into account the EU’s projections of the direct impact of the pandemic, the pandemic will seriously impede the EU’s sustainable development process in the short term. The long-term effects of the pandemic cannot even be outlined, especially at the level of the individual member states. Nevertheless, the effectiveness of the Recovery and Resilience Facility as a key instrument of recovery and of national recovery and resilience plans, will play a decisive role in minimizing or even neutralizing the negative longer-term effects of the COVID-19 pandemic (Zhou et al. [26]). From the perspective of fintech development, this paper tries to construct a comprehensive index to evaluate the green growth of regional economy based on the in-depth analysis of the influence mechanism of green finance on green growth. At the same time, China’s provincial panel data from 2011 to 2018 are selected to test the impact of fintech innovation and green finance on green growth, and its mechanism. It turns out that fintech and green finance significantly promotes green economic growth. 

### 2.2. Willingness and Behavior to Use Digital Payments

Previous studies have focused on the willingness to use and behavior of digital payment. Based on the technology acceptance model (TAM), some scholars have conducted empirical studies on the impact of consumers’ use of mobile payments in Taiwan by adding two factors: perceived credibility, and perceived self-efficacy [1]. Some scholars also divided the research objects into early and late users of mobile payment. Relevant data analysis results show that early users pay more attention to perceived ease of use because they are more innovative and confident, while late users pursue practicality and convenience of mobile payment [2]. Schierz et al. [27] studied the mobile payment behavior of German consumers and confirmed that accessibility, convenience, and individual mobility are the main factors influencing consumers’ use of mobile payment. Similarly, the same conclusion has been reached in the research on the mobile payment behavior of Korean consumers [2]. Other studies have shown that age, external influences, usefulness, and perceived risk are determinants of mobile payment willingness [4]. Based on the model of consumer payment choice, it is found that consumers tend to use cash because it is easy to use and widely accepted. However, the impact of credit card incentive schemes will not only lead to cash substitution, but also lead to debit card substitution. After controlling for the acceptance of merchant cards, consumers still prefer to use cash in many transactions [28]. By examining the impact of debit card use on cash holdings and cash usage, the results show that the payment function of debit cards significantly reduces cash holdings and cash usage, so debit cards can be considered a perfect substitute for cash [29]. Empirical results show that perceived risk is the most important factor in determining whether users use near field communication (NFC) [30]. The structural equation model (SEM) and customer data of 412 restaurants were used to analyze the willingness of north American restaurant customers to use NFC, and the unpopularity of NFC was explained by combining UTAUT and TAM models [6]. In addition, trust and perceived risk were incorporated into the TAM model and factors such as user gender and age were analyzed to find a negative correlation between perceived risk and user willingness to use. The biggest revelation from these results is that psychological factors are an extremely important perspective in studying the operational behavior of mobile payments [31]. However, emotion has been seriously ignored in previous relevant studies, and the influence of emotional factors on the willingness to use electronic payment has been mainly investigated. Park et al. [32] proposed a research model based on the psychological account theory and found that attitude has a positive impact on the intention to use electronic payment. While Verkijika [33] draws on social cognition theory (SCT) and regret theory and points out that influence and expected regret have a significant positive influence on the behavioral intention to adopt mobile payment.

Beck et al. [7] studied the effects of a payment technology innovation (mobile money) on entrepreneurship and economic development in a quantitative dynamic general equilibrium model. In the model, mobile money dominates fiat money as a medium of exchange, since it avoids the risk of theft, but comes with electronic transaction costs. They showed that entrepreneurs with higher productivity and access to trade credit are more likely to adopt mobile money as a payment instrument. Quantitative analysis of adoption rates in various countries shows that institutional and economic factors play different roles in the adoption and use of mobile money innovations [9]. The analysis based on the two-sided market theory shows that the market power of electronic payment network plays an important role in explaining slow absorption and asymmetric price changes [34]. Experimental studies have also shown that in developing countries, cash payments and non-monetary payments have significant uncertainties in the payment process [35]. Although electronic payment methods expand capital mobility, increase trade and interpersonal communication, they pose new challenges in terms of security and privacy, thus influencing the public’s willingness to use electronic payment [36]. Studies in Korea have shown that consumers prefer bank cards to mobile and biometric payments, even though they show the same level of relevant attributes, and consumers’ positive attitude or innovative attitude towards high-tech products exerts a significant positive impact on alternative payment preference [37].

### 2.3. Consumption Behavior and Consumption Demand

According to behavioral life cycle consumption theory, consumers pay more attention to the source, form, and change of income than to the final value of income. It includes three aspects: first, how to use the theory of psychological account to solve the problem of incomplete substitutability between incomes; second, how consumers use the prospect theory to calculate the income under each account; and the third is to use the theory of selective aggregation to investigate the frequency of income accounting by consumers [38]. After that, some scholars constructed the dynamic inconsistencies and hyperbolic discounting model of consumer preference, and used the hyperbolic discounting model to replace the power function discounting model in the standard form. The hyperbolic discount model assumes that consumers are more inclined to current consumption, that is, people always assign relatively large weights to current utility and relatively small weights to future utility, which is more consistent with empirical facts and can better describe consumers’ inconsistent time preference [39,40]. Ludvigson’s [41] empirical research showed that loosening consumer credit will increase liquidity and thus consumer demand. Gross and Souleles [11] showed that credit cards stimulate consumption and increasing consumers’ credit limit will significantly increase consumers’ credit card debt. Oman and Cheema [42] found that for young and less educated consumers, an increase in credit limits had a greater incentive to consume. Zhao and Hsu [43] provided a fundamental study of China’s consumption and output fluctuations. The most recent literature reports that, in the post-1978 period, detrended consumption is significantly more volatile than detrended output in China. Zhao et al. [44], using the Urban Household Survey (UHS) data, produced empirical results demonstrating that consumption inequality in urban China increased by 67% during the sample period and was much larger than 36%, which was obtained directly from the reported raw data. Alexandros et al.’s [45] study found that the estimated value of the subjective discount factor was almost half that of the estimated or calibrated parameter in the empirical literature. This suggests that credit-constrained households tend to be more patient with consumer demand in the event of future debt defaults. Cao et al. [46] estimated separate functions for urban and rural households using household expenditure data and detailed commodity prices (1995 2006). This allows future analysis of social welfare and inequality based on consumption to supplement existing studies based on income.

There are also studies of consumer behavior. Cachon and Swinney [47] concluded that fast fashion systems can be of significant value, particularly when consumers exhibit strategic behavior. Kraft and Munk’s [48] study on Optimal Housing, Consumption, and Investment Decisions over the Life Cycle, derived explicit solutions to life-cycle utility maximization problems involving stock and bond investment, perishable consumption, and the rental and ownership of residential real estate. Hwang and Park [49] presented an empirical study of the impact of Walmart supercenter conversion on consumer shopping behavior, and findings showed that consumers may benefit from reduced shopping costs by making fewer overall trips and increasing their Walmart basket sizes. Jinkins [50] compared the differences in conspicuous consumption motivations between the United States and China, and how conspicuous consumption motivation affects taxation in these countries. Yamamori et al.’s [51] results showed that optimal consumption depends only on real terms instead of nominal terms, and the magnitude of the deviation from optimal consumption in all periods is significantly high, with the large price-fluctuation treatment highest, followed by the small price-fluctuation treatment and then the control treatment. Aviv et al. [52] studied the potential benefits of responsive pricing and demand learning to sellers of seasonal fashion goods, and results demonstrated that the benefits of responsive pricing, in comparison with a benchmark case of a fixed-price policy, depend sharply on the nature of the consumer’s behavior. Iviane et al. [53] compared the factors that determine consumer acceptance SMS (Short Message Service), NFC (Near Field Communication), and QR (Quick Response) mobile payment systems, in addition to determining the principal factors which influence the adoption of these mobile payment systems as means payment. Sugden et al. [54] demonstrated that time-limited offers can have a significant impact on consumer behavior. Petach [55] studied a series of growth models in which households’ preferences display ‘jealousy’ or ‘external habits’: a negative dependence on average consumption. They argue that accounting for consumption externalities in growth models requires consideration of both their static and dynamic effects. 

### 2.4. The Impact of Digital Payments on Consumer Demand

At present, related studies abroad mainly study the driving effect of digital payment on consumption from an empirical perspective. It is believed that consumers will make purchasing decisions from a longer-term perspective. Although consumer credit can solve credit constraints in the short term, its role is limited because it will bring the increase of future debt in the long run [11]. Through the research on the survey data of SCF, it was found that the outstanding balance of credit cards in the United States increased greatly from 1970 to 2000, and credit cards to some extent replaced installment products. Although consumers welcomed the convenience brought by credit cards, they were also worried about the increasing liabilities [12]. The driving effect of electronic payment on consumption and GDP is mainly reflected in that electronic payment makes it more convenient and efficient to purchase, makes it easier for people to get consumer credit, and can obtain more financial resources and actively participate in the digital economy. At the same time, it makes transactions more secure and transparent, thus enhancing mutual trust between the parties and promoting consumption [13]. However, some research shows that electronic payment has no obvious effect on consumption. There is a long-term stable and causal relationship between cash and bank cards, mobile payment, and consumption, while mobile payment has a significant substitution effect on traditional currencies and bank cards [15]. Other studies have shown that vulnerable consumers may face barriers to the use of electronic payments, especially among rural household consumers, few members’ accounts can accept payment, so these families are the slowest to overpay to electronic payments [16].

To sum up, the existing relevant studies have made significant progress in many aspects and many valuable achievements, which are also the important basis of this study, but there are still some shortcomings: (1) most of the existing studies have focused on the impact of traditional factors on consumer demand, while there are relatively few studies on the impact of digital payment on consumer demand; (2) among the few studies on the impact of digital payment on consumption, although some empirical research results support that digital payment means such as credit card will promote consumption demand, and some studies discuss the mechanism of digital payment on consumption, these studies rarely discuss and test the regional differences, urban-rural differences, and cyclical characteristics of the impact of digital payment on consumer demand, which objectively exists; and (3) at present, there is more research on electronic payment tools such as bank card payment, third-party payment, and mobile payment, but there is relatively little research on digital payment, especially on the impact of digital payment on consumer demand and sustainable economic development. Therefore, under the background of COVID-19’s impact on the global economy, this paper studies the impact of digital payment on consumer demand from the perspective of sustainable economic development, and tests the correlation between digital payment and consumer demand. At the same time, through the heterogeneity test, this paper compares and analyzes the regional differences, urban-rural differences, and stage characteristics of the impact of digital payment on consumer demand. Finally, it puts forward the future development of digital payment in China, as well as policy recommendations for digital payment to promote consumer demand and sustainable economic development.

## 3. Research Design

In order to further test the correlation between digital payment and consumer demand, we constructed an econometric model and select relevant data and variables for empirical testing.

### 3.1. Data Source

In view of the availability of data and the timeliness of this study, this paper selects the annual data of all Chinese provinces from 2011 to 2020 as sample data, including 30 Chinese provinces, municipalities, and autonomous regions (excluding Tibet). All data are from Wind database and collated unless otherwise noted.

### 3.2. Variable Selection

#### 3.2.1. Explained Variable

Consumer, Aggregate Consumer Demand. In this paper, per capita consumption expenditure is used instead. Since the current payment methods are mainly cash and digital payment, the consumption demand here refers to the consumption demand realized by cash or digital payment, so this paper selects the per capita consumption expenditure of residents to measure the consumption demand. At the same time, in the process of empirical test, we also take urban consumption demand and rural consumption demand as substitute variables to test.

#### 3.2.2. Core Explanatory Variable

The core explanatory variable of this paper is digital payment (D-pay, Electron Payment), which is an important part of the e-commerce system. The rapid development of digital payment reduces the transaction cost and time cost of financial services, which in turn improves the payment efficiency of residents’ consumption, thus promoting consumer demand and sustainable economic development. Therefore, this paper uses the digital payment index subordinate to the Depth Index to measure the development level of digital payment. The data comes from the Digital Inclusive Finance Index of Peking University [56].

#### 3.2.3. Control Variables

(1) Gross Domestic Product (GDP). As the level of regional economic development is a macro-environmental factor, it will have a direct impact on consumer demand. Therefore, this paper uses the per capita GDP of each province to measure the regional economic development level.

(2) Finance, Financial Development Degree. The development of the financial industry plays an important role in promoting the development of digital payment. The higher the development level of the financial industry in a region, the more active digital payment will be in the region, and the greater the impact on consumer demand will be. Therefore, this paper uses the proportion of financial industry in GDP to measure the development level of the financial industry in each province.

(3) Urbanization Rate. As cities have more network infrastructure and digital payment scenarios than rural areas, the use of digital payment will be greater in areas with a high urbanization rate, thus promoting consumer demand. This paper measures the urbanization rate by the ratio of urban population to total population in each province.

(4) Education, Degree of Education. The more educated people are, the more receptive they are to digital payment, so digital payment can play a bigger role in driving consumer demand. This paper selects the number of higher education students per 10,000 people in each province to measure the educational level of each region.

#### 3.2.4. Instrumental Variables

The level of financial digitalization (Digital) is closely related to the development of Digital payment, which has no direct relationship with residents’ consumption. It plays an important role in supporting the development of Digital payment, but does not directly affect consumption, so it meets the selection criteria of instrumental variables. Therefore, the degree of financial digitization is chosen as the instrumental variable of digital payment development.

In order to be more intuitive, a variable scale is made, as shown in Table 1.

### 3.3. Econometric Model

According to the above analysis, it can be preliminarily determined that there may be a correlation between digital payment and consumer demand, but it is not clear whether there is a clear correlation between variables, and how digital payment affects consumer demand. Therefore, this paper adopts the short panel fixed effect model to test the influence of digital payment on consumer demand, and the regression model is as follows:

(1) $$Consumer_{i,t}=\alpha_{0}+\alpha_{1}Dpay_{i,t}+\alpha_{2}Controls_{i,t}+\varphi_{i}+\varepsilon_{i,t}$$

where subscripts *i* and *t* represent provinces and time. $Consumer_{i,t}$ is the consumption demand of explained variable; $Dpay_{i,t}$ is the core explanatory variable digital payment; $Controls_{i,t}$ represents other control variables that may influence consumption demand; $\varphi_{i}$ represents fixed effect of province; $\varepsilon_{i,t}$ is the stochastic disturbance.

## 4. Test Results and Analysis

### 4.1. Descriptive Statistics

Table 2 presents the descriptive statistical results of each variable. It can be seen from Table 2 that the mean, median, and standard deviation of Consumer are 13.34, 13.31, and 0.45, respectively. The mean, median, and standard deviation of Dpay were 1.85, 1.97, and 0.92, respectively. The mean, median, and standard deviation of GDP were 10.78, 10.73, and 0.44, respectively. The mean, median, and standard deviation of Finance were 9.27, 6.48, and 14.77, respectively. The mean, median, and standard deviation of Urbanization were 0.58, 0.57, and 0.13 respectively. The mean, median, and standard deviation of Education were 1.97, 1.911, and 0.56, respectively. The mean of Digital was 2.90, the median was 3.23, and the standard deviation was 1.17. From the standard deviation of each variable, the standard deviation of most variables is less than 2, indicating that the internal differences of each variable are small. This also shows that the sample data basically present a quasi-normal distribution, and the quality of data selection is high, which can better fit the empirical model and is beneficial to the following regression analysis.

### 4.2. Correlation Test

Correlation analysis refers to the analysis of two or more variable elements with correlation, so as to measure the degree of correlation between two variable factors. For independent variables, control variables, and dependent variables have a certain correlation, can be put into the regression equation, equivalent to the preliminary screening of variables and preliminary judgment of correlation. Therefore, it is necessary to conduct a correlation test, using the Pearson correlation coefficient method. The test results are shown in Table 3. The results show that each variable has a significant impact on the dependent variable. Among them, Dpay and Consumer have a significant correlation, with a correlation coefficient of 0.623 units.

### 4.3. Benchmark Regression

In order to determine whether the fixed-effect model or the random effect model should be selected for empirical test, this paper uses the Hausman test. The results of the Hausman test indicate that the fixed-effect model should be used to conduct the benchmark regression test in this paper. In this paper, the individual effect is fixed, and the test results are shown in Table 4. Where column m1 is the test result with only control variables added, and column m2 is the test result with both control variables and independent variables added. The test results of column m2 show that the independent variable digital payment has a significant positive impact on the dependent variable consumption demand, and is significant at the 1% level. It can be seen that as every unit of digital payment (Dpay) increases, the consumer demand will increase by 0.066 units. This shows that with the rapid development of digital payment in China in recent years, it has a significant role in promoting consumer demand, which is consistent with the previous analysis and expectations.

### 4.4. Endogeneity Test

There may be two endogenous problems in this paper. On the one hand, there is a two-way causality between explanatory variables. Digital payment may stimulate consumption demand, while the increase of consumption demand may also increase people’s daily digital payment, so there is a certain two-way causality. On the other hand, some unobservable factors that affect digital payment and consumer demand at the same time may be omitted. For example, the development of e-commerce may promote the development of people’s consumer demand and digital payment at the same time, resulting in biased inconsistent estimates of regression coefficients. In this regard, we take the following methods to alleviate endogeneity as much as possible.

#### 4.4.1. Variable Lag

For the core variable digital payment and all control variables, lagged one-period values are used, to exclude the influence of the current period and partially eliminate the endogeneity problems caused by reverse causality. The test results are shown in Table 5. It can be seen from Table 5 that the independent variable digital payment (Dpay) has a significant impact on the dependent variable consumer demand, and the consumer demand increases by 0.085 units when the lag period of digital payment (Dpay) increases by one unit. Therefore, it alleviates the endogeneity problem between digital payment and consumer demand to a certain extent.

#### 4.4.2. Instrumental Variable Method

The degree of financial digitization is closely related to the development of digital payment, but has no direct relationship with residents’ consumption. Therefore, the degree of financial digitization can be selected as the instrumental variable of the impact of digital payment on consumer demand. In this paper, the degree of digitalization is used as a variable, taking the instrumental variable method, the three model was established, the first model (m1) is a digitized current as instrumental variable, the second model (m2) is takes the digitized lag term as an instrumental variable, the third model (m3) is a digital level of the current and lag issue at the same time as a tool variable. The test results are shown in Table 6. It can be seen from Table 6 that after adopting the instrumental variable method, the independent variable digital payment (Dpay) has a significant impact on the dependent variable consumer, which is significant at the 1% level. In the model m3, the consumer increases by 0.075 units for every unit of digital payment (Dpay). This shows that the instrumental variable method alleviates the endogeneity problem of the model to a certain extent.

#### 4.4.3. PSM-DID Methods

In order to further identify the promotion effect of the development of digital payment on consumer demand, this paper adopts the PSM-DID method and selects the emergence of Wechat Payment as an exogenous event to further alleviate the endogeneity problem. Specifically, this paper divided the whole sample according to the Internet development degree of each province and selected the three provinces with the lowest level of Internet development as the control group, while the three provinces with the highest level of Internet development were the experimental group. There are reasons to believe that the emergence of Wechat payment has a greater impact on provinces with a higher degree of Internet development, while it has a smaller impact on provinces with a lower degree of Internet development. Next, the samples were matched with a propensity score (PSM) to investigate the changes in consumption demand before and after the appearance of exogenous Wechat payment. The results of PSM matching are shown in Table 7. It can be seen from Table 8 that, after matching, the mean value of consumer in the control group is 13.628, and that in the experimental group is 13.501, with a significant difference of 0.127.

#### 4.4.4. PVAR Model

**Figure 1:**
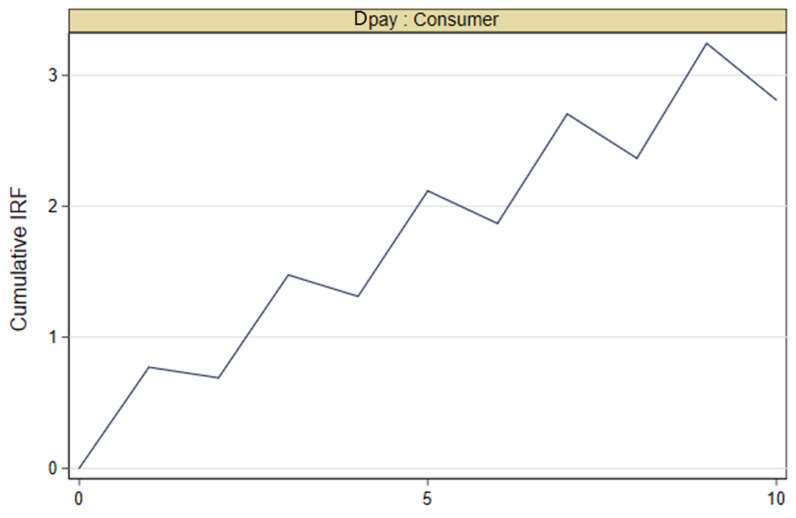
Pulse response diagram.

In order to further dynamically investigate the relationship between digital payment and consumer demand, we assumed that all variables were endogenous variables and used PVAR model for estimation. The test results are shown in Table 8 and Figure 1. As can be seen from Table 8 and Figure 1, digital payment has a cumulative positive impact on consumer demand, which further demonstrates the reliability of the previous conclusion.

### 4.5. Robustness Test

The methods of the robustness test generally include variable substitution method, supplementary variable method, sample regression method, adjusting sample period, changing sample size method, Robust robustness analysis, and so on. In order to test the robustness of the model, this paper used the variable substitution method, changing sample size, and Robust analysis.

#### 4.5.1. Variable Substitution Method

In this paper, the dependent variable consumer is replaced by the total retail sales of social consumer goods. The test results are shown in Table 9. It can be seen from Table 10 that the independent variable Dpay has a significant impact on the dependent variable after replacement. For each unit increase in the lag period of Dpay, consumer2 increases by 0.095 units. This shows that the model is still robust after replacing the dependent variable.

#### 4.5.2. Changing the Sample Size

The test results are shown in Table 11. From Table 10, it can be seen that the independent variable Dpay has a significant impact on the dependent variable consumer. For each additional unit of the core variable Dpay lag, consumer2 will increase by 0.068 units. This further shows that the model constructed in this paper is robust.

#### 4.5.3. Robust Test

Finally, in order to reduce the influence caused by the existence of outliers in data sample points, Robust regression was used to replace the least square method for Robust analysis of variables. The test results are shown in Table 11. As can be seen from Table 12, the independent variable digital payment (Dpay) has a significant impact on the dependent variable consumer demand (Consumer). For every unit increase in the lagging phase of Dpay, the consumer will increase by 0.068 units. This further shows that the model is robust.

### 4.6. Heterogeneity Test

In order to compare the impact of digital payment on residents’ consumption demand in different regions, this paper divides 30 provinces and cities in China (excluding Tibet) into four regions: the East, the middle, the West, and the northeast. The division of the four regions is based on China’s 11th five-year plan. The eastern region includes 10 regions including Beijing, Tianjin, Hebei, Shanghai, Jiangsu, Zhejiang, Fujian, Shandong, Guangdong, and Hainan, the western region includes 12 regions including Inner Mongolia, Guangxi, Chongqing, Sichuan, Guizhou, Yunnan, Tibet, Shaanxi, Gansu, Qinghai, Ningxia, and Xinjiang, and the central region includes Shanxi, Anhui, Jiangxi, Henan, Hubei There are 6 regions in Hunan, and the Northeast includes Liaoning, Jilin, and Heilongjiang.

#### 4.6.1. Regional Heterogeneity

The regression results by region are shown in Table 12. It can be seen from Table 12 that only in the eastern and western regions, digital payment has a significant impact on consumer demand. In the eastern region, for every unit increase in Dpay, 0.046 units of consumer will be added. In the Western region, for every unit increase in Dpay, 0.142 units of consumer were added. In northeast and central China, although digital payment has a positive impact on consumer demand, the impact is not obvious. This shows that with the widespread use of electronic money, electronic money has an obvious substitution effect on traditional cash payment. Due to the relatively developed economy in eastern China and the longer period using digital payment, the substitution effect of digital payment on cash is more obvious in the sample range. Therefore, the promotion effect of digital payment on consumer demand is more obvious. At the same time, due to the digital pay relatively backward western regions for a long time, but in the last 10 years, with the improvement of China’s Internet penetration rate, the financial degree of digitization, financial development, and the popularity of e-commerce platforms to promote the rapid development of the western region’s digital payment, and has thus stimulated the consumption potential. Therefore, the role of digital payment in promoting consumer demand is highlighted. However, in central and northeast China, digital payment has a relatively weak impact on consumer demand.

#### 4.6.2. Temporal Heterogeneity

In order to analyze the difference in the impact of digital payment on consumer demand in different regions at different developmental stages, this paper divides the samples into two stages, 2011–2015 and 2016–2020, according to the division method of China’s 12th Five-Year Plan (2011–2015) and 13th Five-Year Plan (2016–2020). Regression analysis was conducted by region, and the test results are shown in Table 13 and Table 14. As can be seen from Table 14 and Table 15, digital payment in each region has no significant impact on consumer demand in these two stages. This is mainly due to the fact that China’s Third Payment started to issue licenses in 2011, which normalized the Third Payment and greatly promoted the rapid development of the Third Payment and gradually popularized digital payment. Its promoting effect on consumer demand showed a relatively stable trend. Of course, it may also be due to the short sample range selected.

#### 4.6.3. Urban-Rural Heterogeneity

China is a country with unbalanced economic development, not only manifested in regional differences, but also in urban and rural differences. In order to further compare and analyze the difference in the impact of digital payment on consumer demand between urban and rural areas in China, this paper selects con-urban and con-rural per capita consumption expenditure as samples, and replaces the dependent variable per capita consumption expenditure with urban consumption expenditure and rural consumption expenditure. The test results are shown in Table 15. As can be seen from Table 15, when digital payment Dpay increases by one unit, per capita consumption expenditure (con-urban) increases by 0.111 units. For each additional unit of Dpay, the rural per capita consumption expenditure (con rural) will increase by 0.139 units, and the impact of digital payment on rural consumption expenditure is greater than that of urban consumption expenditure. This shows that in the process of digital payment affecting consumption demand, the utility of rural areas is greater than that of cities and towns, which also shows that compared with cities and towns, rural consumption potential is greater.

## 5. Conclusions

This paper is based on the theory of consumer demand, inclusive finance and technology acceptance from the perspective of sustainable economic development. This paper selects panel data of Chinese provinces from 2011 to 2020, selects indicators related to digital payment and consumer demand, constructs an econometric model, tests the correlation between digital payment and consumer demand, reveals the impact of digital payment on consumer demand and sustainable economic development, and puts forward relevant policy suggestions. Through the above theoretical analysis and empirical test, the following basic conclusions are drawn: (1) in the context of the COVID-19 pandemic, digital payment plays a special and very important role in promoting household consumption and sustainable economic development; and (2) the empirical results show that digital payment has a significant impact on consumer demand. When Dpay increases by one unit, consumer demand will increase by 0.066 units. This shows that with the rapid development of digital payment in China in recent years, it plays an obvious role in stimulating consumer demand, thus promoting sustainable economic development. (3) The impact of digital payment on consumer demand has obvious heterogeneity. The heterogeneity test results show that from a regional perspective, in the eastern and western regions, digital payment has a significant positive impact on consumer demand, while in the northeast and central regions, although digital payment has a positive impact on consumer demand, the impact is not obvious. However, in terms of time, the impact of digital payment on consumer demand in various regions is not significant, which may be due to the short sample interval selected. From the perspective of urban-rural differences, whether in urban or rural areas, the impact of digital payment on consumption demand is very obvious, and the impact of digital payment on rural consumption expenditure is greater than that of urban consumption expenditure. This indicates that in the process of digital payment affecting consumer demand, the utility of rural areas is greater than that of urban areas, which also indicates that rural consumption potential is greater than that of urban areas.

Existing studies (e.g., Li et al., 2020) [20] have found that digital financial inclusion can promote household consumption. Online shopping, digital payment, obtaining online credit, and purchasing Internet financial products and commercial insurance are the main intermediary variables of digital finance affecting household consumption. This paper mainly studies the impact of digital payment on consumer demand from the perspective of sustainable economic development, which is a further development of existing research. Therefore, the research of this paper is obviously different from the existing research. First, the purpose of this paper is to study how digital payment can promote consumer demand and sustainable economic development under the background of the global COVID-19 pandemic, which has a serious impact on the economy and faces great challenges to sustainable economic development. The second is to study the impact of digital payment on residents’ consumption demand from the perspective of sustainable economic development, which is relatively new. Third, different from the previous theoretical studies that mostly focus on the influencing factors of consumer demand, this paper pays more attention to empirical analysis, and reveals the main factors and mechanisms of digital payment affecting consumer demand through empirical testing. Fourth, this paper tests the impact of digital payment on urban residents’ consumption, rural residents’ consumption and the per capita consumption of all residents at the same time, and tests and compares the heterogeneity of residents’ consumption in different types, regions and stages. On this basis, this paper draws the following conclusion: in view of the positive impact of digital payment on the consumption of Chinese residents, against the backdrop of the current COVID-19 impact on the global economy and China’s policy of shifting the driving force of economic growth from investment and export to consumption, China should vigorously develop digital payments to promote consumption and sustainable economic development. At present, the development of electronic payment faces many problems. Therefore, it is high time that we should improve relevant laws and regulations to ensure the development of digital payment, establish, and perfect credit investigation systems and standardize digital payment transactions, and accelerate financial technology innovation, promote the development of digital payment, and maintain financial security. Along with these trends, we should give full play to the special role of digital payment in stimulating consumer demand and promoting sustainable economic development. The conclusion of this paper can also be used as a reference for other countries.

Of course, this paper also has shortcomings. First, the sample period selected in this paper is from 2010 to 2020, so it cannot fully discuss and compare the difference in the impact of digital payment on consumer demand during and after the pandemic. Second, the sample size of inter-provincial panel data is relatively small, which will affect the reliability of research conclusions to some extent. Third, there is no in-depth study on the mechanism and path of digital payment’s impact on consumer demand. Fourth, there is a lack of comparison between the impact of digital payment in China and other countries on consumer demand.

In view of the above shortcomings, further research can be carried out from the following directions. First, the sample range can be expanded to several years after the outbreak of COVID-19, so as to better compare the impact of digital payment on consumer demand before and after the pandemic. In terms of research methods, COVID-19 can be regarded as an exogenous shock, and the impact of digital payment on consumer demand and economic sustainability can be tested by the difference in difference (DID) method. Second, micro data can be used to deeply study the influence of digital payment on household consumption and its mechanism, such as being combined with household survey data. The third is to further explore the influence mechanism and path of digital payment on consumer demand, so as to answer the important question of how electronic money affects consumer demand. Fourth, we can do in-depth analysis and make a comparison between China and other countries on the impact of digital payment on consumer demand differences, in order to put forward more general policy recommendations.

## Figures and Tables

**Table 1:** Variable definition table.

Variable Types	Variable Name	Variable Code	Variable Definition and Measurement Method
Explained variable	Consumer demand	Consumer	Per capita consumption expenditure
	Urban consumption demand	Con-urban	Urban per capita consumption expenditure
	Rural consumption demand	Con-rural	Rural per capita consumption expenditure
Explanatory variables	Digital pay	D-pay	Digital payment index in Peking University Digital Financial Inclusion Index
Control variables	Level of economic development	GDP	GDP per capita
	Degree of financial development	Finance	Financial sector as a share of GDP
	Urbanization rate	Urbanization	Ratio of urban population to total population at year-end
	Education level	Education	Number of higher education students per 10,000 people
Instrumental variable	Degree of digitization	Digital	Financial digitization degree index in Peking University Digital Financial Inclusion Index

**Table 2:** Descriptive analysis.

Variables	N	Mean	p50	sd	Min	Max
Consumer	310	13.34	13.31	0.45	12.17	14.67
Con-urban	310	9.93	9.93	0.29	9.34	10.78
Con-rural	310	9.15	9.16	0.38	8.05	10.02
Dpay	310	1.85	1.97	0.92	0.00	3.80
GDP	310	10.78	10.73	0.44	9.68	12.01
Finance	310	9.27	6.48	14.77	0.46	97.53
Urbanization	310	0.58	0.57	0.13	0.22	0.90
Education	310	1.97	1.91	0.56	0.74	4.13
Digital	310	2.90	3.23	1.17	0.08	4.62

**Table 3:** Correlation analysis.

Variable	Consumer	Con-Urban	Con-Rural	Dpay	DigitalL	GDP	Finance	Urbanization	Education
Consumer	1.000								
Con-urban	0.931	1.000							
	0.000								
Con-rural	0.808	0.923	1.000						
	0.000	0.000							
Dpay	0.623	0.818	0.851	1.000					
	0.000	0.000	0.000						
Digital	0.414	0.614	0.657	0.840	1.000				
	0.000	0.000	0.000	0.000					
GDP	0.866	0.896	0.899	0.697	0.451	1.000			
	0.000	0.000	0.000	0.000	0.000				
Finance	−0.036	0.086	−0.110	0.046	0.070	0.014	1.000		
	0.533	0.129	0.054	0.425	0.221	0.803			
Urbanization	0.904	0.789	0.784	0.476	0.259	0.864	−0.224	1.000	
	0.000	0.000	0.000	0.000	0.000	0.000	0.000		
Education	0.477	0.408	0.489	0.347	0.249	0.514	−0.214	0.638	1.000
	0.000	0.000	0.000	0.000	0.000	0.000	0.000	0.000	

**Table 4:** Benchmark regression results.

Variable	(1)	(2)
	m1	m2
	Consumer	Consumer
Dpay		0.066 ***
		(3.698)
GDP	0.408 ***	0.226 ***
	(6.666)	(2.924)
Finance	0.009 ***	0.005 **
	(3.519)	(1.992)
Urbanization	3.539 ***	3.150 ***
	(9.488)	(8.300)
Education	−0.200 ***	−0.162 ***
	(−4.899)	(−3.925)
Constant	7.202 ***	9.221 ***
	(15.015)	(12.811)
Observations	310	310
R-squared	0.884	0.890
Number of id	31	31
F	525.9	442.8

z-statistics in parentheses, *** *p* < 0.01, ** *p* < 0.05

**Table 5:** Endogeneity test: variable lag regression analysis.

Variable	(1)	(2)
	m1	m2
	Consumer	Consumer
L.Dpay		0.085 ***
		(4.558)
L.GDP	0.218 ***	−0.013
	(3.141)	(−0.151)
L.Finance	0.017 ***	0.013 ***
	(5.612)	(4.114)
L.Urbanization	3.602 ***	3.080 ***
	(8.486)	(7.265)
L.Education	−0.158 ***	−0.118 **
	(−2.891)	(−2.209)
Constant	9.116 ***	11.712 ***
	(16.773)	(15.150)
Observations	279	279
R-squared	0.841	0.854
Number of id	31	31
F	322.8	283.3

z-statistics in parentheses, *** *p* < 0.01, ** *p* < 0.05

**Table 6:** Instrumental variable method.

Variable	(1)	(2)	(3)
	m1	m2	m3
	Consumer	Consumer	Consumer
Dpay	0.102 ***	0.074 ***	0.075 ***
	(2.676)	(2.730)	(2.834)
GDP	0.130	0.195 *	0.193 *
	(1.079)	(1.948)	(1.951)
Finance	0.003	0.002	0.002
	(1.044)	(0.594)	(0.581)
Urbanization	2.943 ***	3.047 ***	3.041 ***
	(6.852)	(6.946)	(6.962)
Education	−0.142 ***	−0.159 ***	−0.159 ***
	(−3.093)	(−3.383)	(−3.383)
Constant	10.295 ***	9.632 ***	9.659 ***
	(8.244)	(9.737)	(9.943)
Observations	310	279	279
Number of id	31	31	31
chi2	8.759 × 10^6^	8.213 × 10^6^	8.215 × 10^6^

z-statistics in parentheses, *** *p* < 0.01, * *p* < 0.1.

**Table 7:** PSM analysis results.

Sample	Treated	Controls	Difference	S.E.	Tstat
Unmatched	13.628	13.200	0.428	0.049	8.7
Matched	13.628	13.501	0.127	0.105	1.2

**Table 8:** Analysis results of PVAR model.

Variable	Coef.	Std. Err.	z	P > z	95% Conf.	Interval
Consumer						
L1.Consumer	−2.143	0.920	−2.330	0.020	−3.946	−0.339
L1.Dpay	0.771	0.247	3.130	0.002	0.288	1.254
Dpay						
L1.Consumer	−4.358	1.589	−2.740	0.006	−7.472	−1.243
L1.Dpay	2.038	0.421	4.830	0.000	1.212	2.864

**Table 9:** Robustness analysis: variable substitution method.

Variable	(1)	(2)
	m1	m2
	Consumer2	Consumer2
Dpay		0.095 ***
		(3.065)
GDP	1.049 ***	0.788 ***
	(9.965)	(5.876)
Finance	−0.006	−0.011 **
	(−1.261)	(−2.315)
Urbanization	2.087 ***	1.528 **
	(3.251)	(2.322)
Education	−0.300 ***	−0.245 ***
	(−4.266)	(−3.426)
Constant	6.099 ***	8.999 ***
	(7.390)	(7.213)
Observations	310	310
R-squared	0.807	0.814
Number of id	31	31
F	287.9	239.2

z-statistics in parentheses, *** *p* < 0.01, ** *p* < 0.05.

**Table 10:** Robustness analysis: sample tailing treatment.

Variable	(1)	(2)
	m1	m2
	Consumer	Consumer
Dpay		0.068 ***
		(3.789)
GDP	0.388 ***	0.208 ***
	(6.422)	(2.751)
Finance	0.007 ***	0.003
	(2.759)	(1.193)
Urbanization	3.724 ***	3.237 ***
	(9.766)	(8.216)
Education	−0.215 ***	−0.167 ***
	(−4.991)	(−3.802)
Constant	7.357 ***	9.392 ***
	(15.548)	(13.254)
Observations	310	310
R-squared	0.885	0.891
Number of id	31	31
F	529.1	446.7

z-statistics in parentheses, *** *p* < 0.01.

**Table 11:** Robustness analysis: Robust regression.

Variable	(1)	(2)
	m1	m2
	Consumer	Consumer
Dpay		0.068 **
		(2.392)
GDP	0.388 ***	0.208
	(3.250)	(1.414)
Finance	0.007	0.003
	(0.902)	(0.513)
Urbanization	3.724 ***	3.237 ***
	(5.089)	(4.585)
Education	−0.215 ***	−0.167 **
	(−2.904)	(−2.215)
Constant	7.357 ***	9.392 ***
	(7.802)	(7.031)
Observations	310	310
R-squared	0.885	0.891
Number of id	31	31
F	163.9	126.4

z-statistics in parentheses, *** *p* < 0.01, ** *p* < 0.05.

**Table 12:** Regional heterogeneity test.

Variable	(1)	(2)	(3)	(4)
	Northeast	East	Central	West
	Consumer	Consumer	Consumer	Consumer
Dpay	0.056	0.046 *	0.080	0.142 ***
	(0.981)	(1.745)	(0.900)	(2.640)
GDP	0.425	0.142	0.014	0.272 *
	(1.373)	(1.278)	(0.058)	(1.712)
Finance	0.023	0.025 ***	0.006	0.001
	(1.133)	(3.410)	(0.193)	(0.231)
Urbanization	2.423	2.792 ***	4.234	2.244 *
	(0.760)	(6.167)	(1.370)	(1.982)
Education	−0.379 *	0.001	−0.238	−0.257 ***
	(−2.072)	(0.014)	(−1.110)	(−3.091)
Constant	8.019 **	9.896 ***	10.949 ***	9.359 ***
	(2.759)	(8.781)	(6.395)	(6.574)
Observations	30	100	60	120
R-squared	0.864	0.928	0.907	0.892
Number of id	3	10	6	12
F	27.93	220.2	95.50	170.4

z-statistics in parentheses, *** *p* < 0.01, ** *p* < 0.05, * *p* < 0.1.

**Table 13:** Time heterogeneity test: 2010–2015.

Variable	(1)	(2)	(3)	(4)
	Northeast	East	Central	West
	Consumer	Consumer	Consumer	Consumer
Dpay	0.152	−0.012	−0.101	0.066
	(1.660)	(−0.383)	(−1.234)	(1.270)
GDP	1.096 **	0.178	0.114	0.145
	(3.120)	(0.704)	(0.449)	(0.996)
Finance	−0.023	0.033 ***	0.079 ***	0.007 **
	(−0.570)	(2.773)	(2.919)	(2.042)
Urbanization	8.088	4.398 ***	6.597 *	3.536 **
	(1.875)	(5.337)	(1.940)	(2.553)
Education	−1.849 **	0.010	−0.314	0.042
	(−2.586)	(0.091)	(−1.110)	(0.234)
Constant	0.825	8.444 ***	8.762 ***	9.605 ***
	(0.205)	(3.667)	(4.634)	(7.718)
Observations	15	50	30	60
R-squared	0.926	0.928	0.949	0.942
Number of id	3	10	6	12
F	17.43	89.76	70.84	139.1

z-statistics in parentheses, *** *p* < 0.01, ** *p* < 0.05, * *p* < 0.1.

**Table 14:** Time heterogeneity test: 2016–2020.

Variable	(1)	(2)	(3)	(4)
	Northeast	East	Central	West
	Consumer	Consumer	Consumer	Consumer
Dpay	−0.022	−0.123	0.078	0.128
	(−0.036)	(−0.798)	(0.375)	(0.773)
GDP	0.488	0.702 **	0.110	0.958 **
	(0.353)	(2.108)	(0.180)	(2.406)
Finance	0.131 **	0.013	−0.059	0.000
	(2.398)	(1.109)	(−0.968)	(0.047)
Urbanization	−2.996	2.462 *	4.566	−3.352
	(−0.303)	(1.890)	(0.976)	(−1.176)
Education	−0.423	−0.146	−0.389	−0.066
	(−1.233)	(−1.303)	(−1.304)	(−0.478)
Constant	10.402	4.750	10.423 **	4.708
	(1.034)	(1.537)	(2.133)	(1.460)
Observations	15	50	30	60
R-squared	0.675	0.693	0.519	0.463
Number of id	3	10	6	12
F	2.907	15.81	4.101	7.421

z-statistics in parentheses, ** *p* < 0.05, * *p* < 0.1.

**Table 15:** Urban and rural heterogeneity test.

Variable	(1)	(2)	(3)
	m1	m2	m3
	Consumer	Con-urban	Con-rural
Dpay	0.066 ***	0.111 ***	0.139 ***
	(3.698)	(12.003)	(11.998)
GDP	0.226 ***	0.313 ***	0.518 ***
	(2.924)	(7.830)	(10.391)
Finance	0.005 **	0.002	0.001
	(1.992)	(1.520)	(0.618)
Urbanization	3.150 ***	0.827 ***	0.551 **
	(8.300)	(4.213)	(2.252)
Education	−0.162 ***	−0.008	0.096 ***
	(−3.925)	(−0.383)	(3.622)
Constant	9.221 ***	5.860 ***	2.791 ***
	(12.811)	(15.745)	(6.019)
Observations	310	310	310
R-squared	0.890	0.963	0.970
Number of id	31	31	31
F	442.8	1444	1757

z-statistics in parentheses, *** *p* < 0.01, ** *p* < 0.05.

## Data Availability

The data used to support the findings of this study are available from the corresponding author upon request.
